# Coleoptera claws and trichome interlocking

**DOI:** 10.1007/s00359-022-01554-1

**Published:** 2022-05-26

**Authors:** Gianandrea Salerno, Manuela Rebora, Silvana Piersanti, Valerio Saitta, Elena Gorb, Stanislav Gorb

**Affiliations:** 1 Dipartimento di Scienze Agrarie, Alimentari e Ambientali, University of Perugia, Borgo XX Giugno, 74, 06121 Perugia, Italy; 2 Dipartimento di Chimica, Biologia e Biotecnologie, University of Perugia, Via Elce di Sotto, 8, 06123 Perugia, Italy; 3 Department of Functional Morphology and Biomechanics, Zoological Institute, Kiel University, Am Botanischen Garten 9, 24098 Kiel, Germany

**Keywords:** Insect attachment, Ladybird, Hairy leaves, Friction, Leaf replicas

## Abstract

The present study tests the hypothesis that the specialized claws with a basal tooth found in some coccinellid beetles represent an adaptation to interlock with flexible unbranched trichomes of different plants. We compared the attachment ability of three Coleoptera species, *Chnootriba elaterii, Harmonia axyridis* (both Coleoptera: Coccinellidae), and *Chrysolina herbacea* (Coleoptera: Chrysomelidae) with claws of different shape. The attachment ability of insect individuals with or without claws to a plant with leaves bearing straight non-branched trichomes (*Cucurbita moschata*) and to a plant with smooth leaves (*Prunus laurocerasus*) was measured in traction force experiments. Insect attachment ability was also tested on a resin replica of *C. moschata* leaf, to variate trichome stiffness, and on glass as a reference surface. Centrifugal force tester experiments were performed to compare the attachment ability of the two ladybird species to glass and to the leaf of *C. moschata*. Natural and artificial substrates were characterized in cryo-SEM. The collected data reveal that plant trichomes can enhance insect attachment to plant surface compared with smooth glass by increasing insect friction force, but this is directly related to the trichome stiffness. To effectively grasp soft trichomes, insects evolved special claws-associated structures, such as the dentate claws observed in Coccinellidae.

## Introduction

To adhere successfully to a substrate, insects developed various types of leg attachment devices typically located on their tarsi (Gorb 2001). Hairy pads or smooth flexible pads (arolia, pulvilli, and euplantulae) are mainly used on smooth and microrough surfaces (Beutel and Gorb 2001), while pretarsal claws are used to interlock with rough surfaces, when the distances between adjacent asperities are larger than the claw tip diameter (Dai et al. 2002). Claws and pads are constituted of the cuticle with different chemical composition, since sclerotised chitinous material increases claw stiffness, while a high proportion of the elastomeric protein resilin makes insect attachment pads elastic and soft (Michels et al. 2016; Rebora et al. 2018, 2021; Zhang et al. 2019; Salerno et al. 2020a).

Insect claws show different designs according to the insect biology as adaptations to particular substrates and can be more or less developed (e.g., Friedemann et al. 2014a, b). In adult insects, they are typically paired structures, but very seldom only a single claw can be present (Gorb 2001). Interesting adaptations are present in the claws of phoretic and parasitic insects showing specialized claws, whose morphology allows a strong attachment to the host feathers (Petersen et al. 2018; Liu et al. 2019) or hairs (Büscher et al. 2021).

During the long antagonistic co-evolution between phytophagous insects and host plants, the latter have evolved a variety of features to protect themselves against herbivores. Among other specializations, mechanical barriers represented by epicuticular waxes, spines, or trichomes (Jeffree 1986; Southwood 1986; Gorb and Gorb 2013) can reduce insect locomotion performance and attachment on the plant surface. Phytophagous insects and predatory insects feeding on them, on the other hand, have evolved attachment structures to overcome these barriers. Leaf trichomes, which can be glandular or non-glandular, branched or non-branched, and unicellular or multicellular (Southwood 1986), affect patterns of insect herbivory and insect abundance for a variety of plant species (reviewed in Levin 1973; Johnson 1975). For example, the glabrous morph of the perennial herb *Arabidopsis lyrata* (L.) O'Kane & Al-Shehbaz (Brassicaceae) is more damaged by phytophagous insects than the trichome-bearing morph (Løe et al. 2007). Also, some plant species respond to damage caused by herbivores by producing new leaves with an increased density and/or number of trichomes (Dalin et al. 2008). Plant glandular trichomes may additionally secrete sticky [e.g., in Solanum spp. (Solanaceae), Gibson 1971] or poisonous chemicals challenging herbivorous insects (Duffey 1986), while non-glandular trichomes, such as hooked trichomes of *Phaseolus vulgaris* L. (Fabaceae), may impale and entrap insects (Richardson 1943; Johnson 1953; Pillemer and Tingey 1976, 1978; Szyndler et al. 2013; Xing et al. 2017; Rebora et al. 2020a). The presence of large trichomes, such as those of *S. melongena* L. (Solanaceae), can reduce ladybird egg adhesion (Salerno et al. 2021). A dense pubescence on the plant surface can impede insect locomotion or leaf feeding, especially in insects with a high degree of contact with the leaf surface, such as caterpillars (Lill et al. 2006; Agrawal et al. 2009). Generally, adult insect performance is affected less negatively (or even positively) by the presence of trichomes, as shown in different lepidopteran species feeding on the soybean, where the leaf pubescence functions as a resistance mechanism against larvae, but enhances adult oviposition (Lambert et al. 1992).

A positive effect of trichomes in enhancing insect locomotion has been described also in long-legged sucking insects, such as Hemiptera. This is the case in the generalist *Nezara viridula* (L.) (Hemiptera: Pentatomidae), which was able to realize a stronger attachment force on the non-glandular stellate trichomes of *S. melongena* leaf than on smooth glass (Salerno et al. 2018). For this species, it was hypothesized that non-glandular stellate trichomes with vertical arms and accumbent side arms could be used by insect claws to improve attachment during pulling. A very good performance on hairy surfaces was demonstrated also by the omnivorous mirid bug *Dicyphus erran*s (Wolff) (Hemiptera: Miridae) that typically lives on pubescent plants (Voigt et al. 2007). It shows morphological and behavioral adaptations to hairy plants resulting in higher predation, fecundity, and attachment ability, if compared to plants without trichomes. Observations in scanning electron microscopy revealed that sharpened claws grasp the smooth trichome surface to get a grip, while pseudopulvilli are not involved (Voigt et al. 2007). Regarding Coleoptera, inhibition of movements was described in coccinellid larvae on plant surfaces with trichomes (Belcher and Thurston 1982), but the attachment of the coccinellid *Serangium japonicum* Chapin (Coleoptera: Coccinellidae) at the adult stage was very high on the dense stellate trichomes of *S. melongena* and still good on *Cucumis sativus* L. (Cucurbitaceae) leaves covered by cone-shaped trichomes (Yao et al. 2021). It was hypothesized that the claw groove of *S. japonicum* could fit well to the width of the abundant side arms of trichomes. Behavioral experiments with *Chrysolina fastuosa* Scopoli (Coleoptera: Chrysomelidae) showed that these insects were unable to attach to plant substrates covered with a wax bloom (Gorb and Gorb 2002). However, in cases of a complex surface coverage (wax bloom and trichomes together), such as on the lower leaf surface in *Aquilegia vulgaris* L. (Ranunculaceae), *Robinia pseudoacacia* L., and *Trifolium montanum* L. (both Fabaceae), beetles demonstrated quite normal attachment. Trichomes here may provide anchorage sites for insect claws.

In the present investigation, we test the hypothesis that claws with different shapes (bifid dentate, dentate, or divergent) observed in the adult stage of three different Coleoptera species represented by the phytophagous ladybird *Chnootriba elaterii* (Rossi) (Coleoptera: Coccinellidae), the predaceous ladybird *Harmonia axyridis* (Pallas) (Coleoptera: Coccinellidae), and the phytophagous *Chrysolina herbace*a (Duftschmid) (Coleoptera: Chrysomelidae) could have different involvement in insect attachment to hairy surfaces. In particular, we measured the attachment ability of insect individuals with or without claws to the plant with hairy leaves covered by straight non-branched trichomes [*Cucurbita moschata* Duchesne ex Poir. (Cucurbitaceae)] and to the plant with smooth leaves [*Prunus laurocerasus* L. (Rosaceae)]. Insect attachment was measured also on glass and on a resin replica of the *C. moschata* leaf to remove a possible effect of glandular trichomes and to variate the trichome stiffness. Observations in the cryo-scanning electron microscope were performed to describe the shape of the insect claws and to structurally characterize the natural and artificial surfaces under consideration.

## Materials and methods

### Insects

**Fig. 1:**
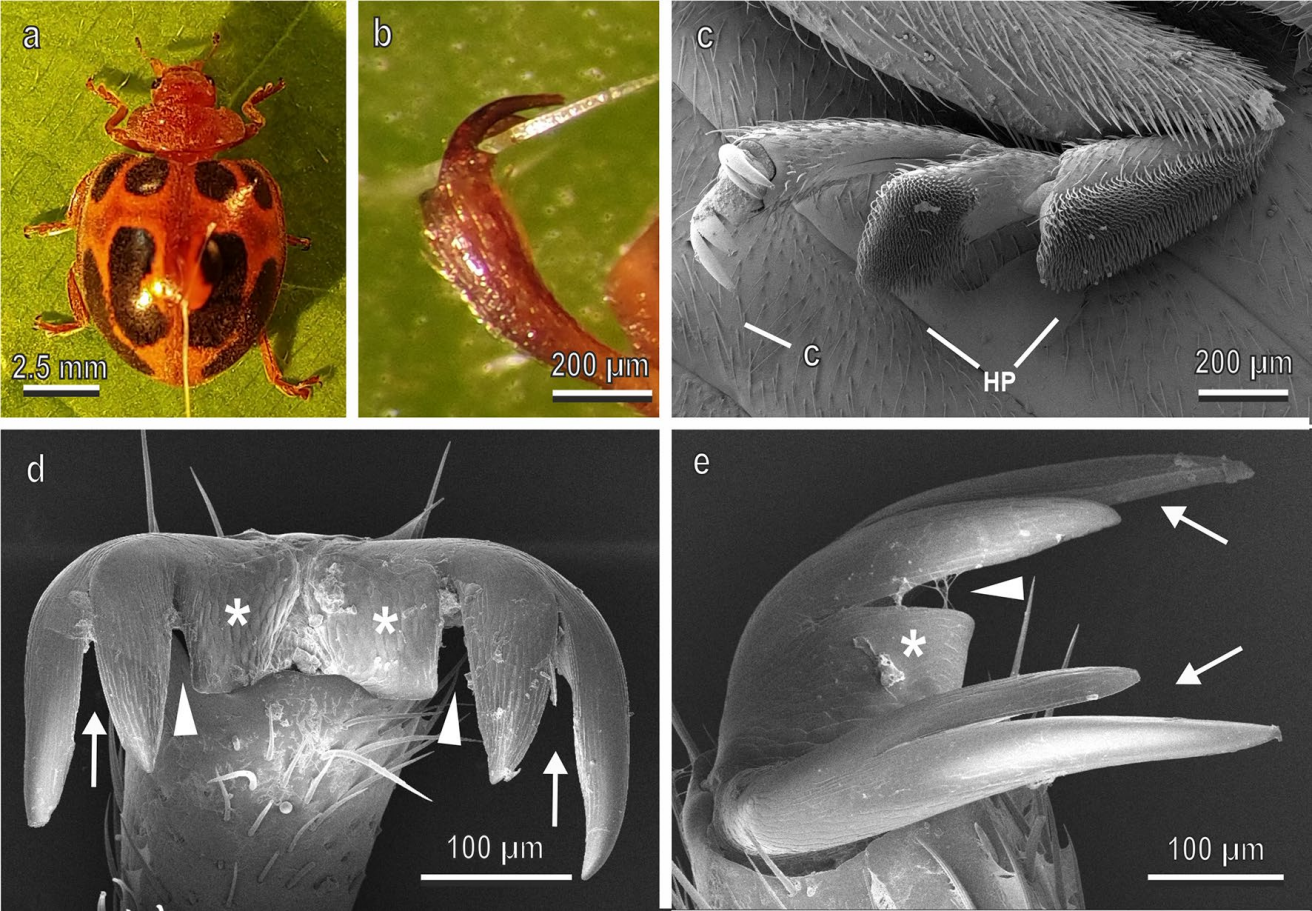
Female of *Chnootriba elaterii* under stereomicroscope (**a**, **b**) and tarsal attachment devices in the cryo-SEM (**c**–**e**). **a**, Insect attached to the force sensor by means of the polyethylene thread; **b**, details of the interlocking between the claws and a single trichome of *Cucurbita moschata* leaf; **c**, latero-ventral view of the tarsus with the attachment organs represented by a pair of pretarsal bifid dentate claws (C) and two hairy pads (HP) situated on the ventral side of the first and second tarsal segments; **d**,**e**, details of the pair of claws in frontal (**d**) and lateral (**d**) view. Each claw is bifid (arrows) and has a basal tooth (asterisk) separated from the claw by a deep cleft (arrow head)

**Fig. 2:**
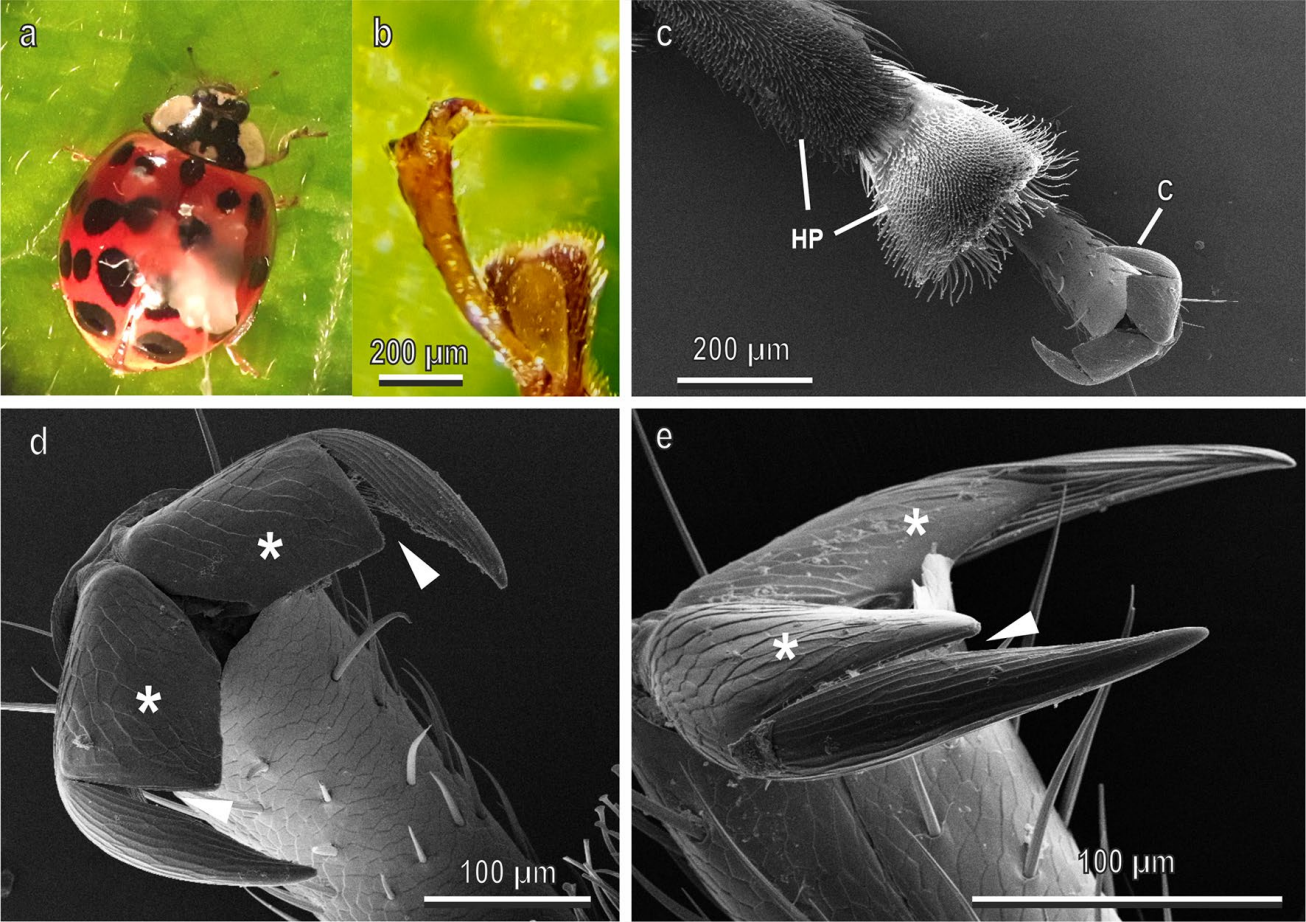
Female of *Harmonia axyridis* under stereomicroscope (**a**, **b**) and tarsal attachment devices in the cryo-SEM (**c**–**e**). **a**, Insect attached to the force sensor by means of the polyethylene thread; **b**, details of the interlocking between the claws and a single trichome of *Cucurbita moschata* leaf; **c**, ventral view of the tarsus with the attachment organs represented by a pair of pretarsal dentate claws (**C**) and two hairy pads (HP) situated on the ventral side of the first and second tarsal segments; **d**, **e**, details of the pair of claws in frontal (**d**) and lateral (**d**) view. Each claw has a basal tooth (asterisk) separated from the claw by a deep cleft (arrow head)

**Fig. 3:**
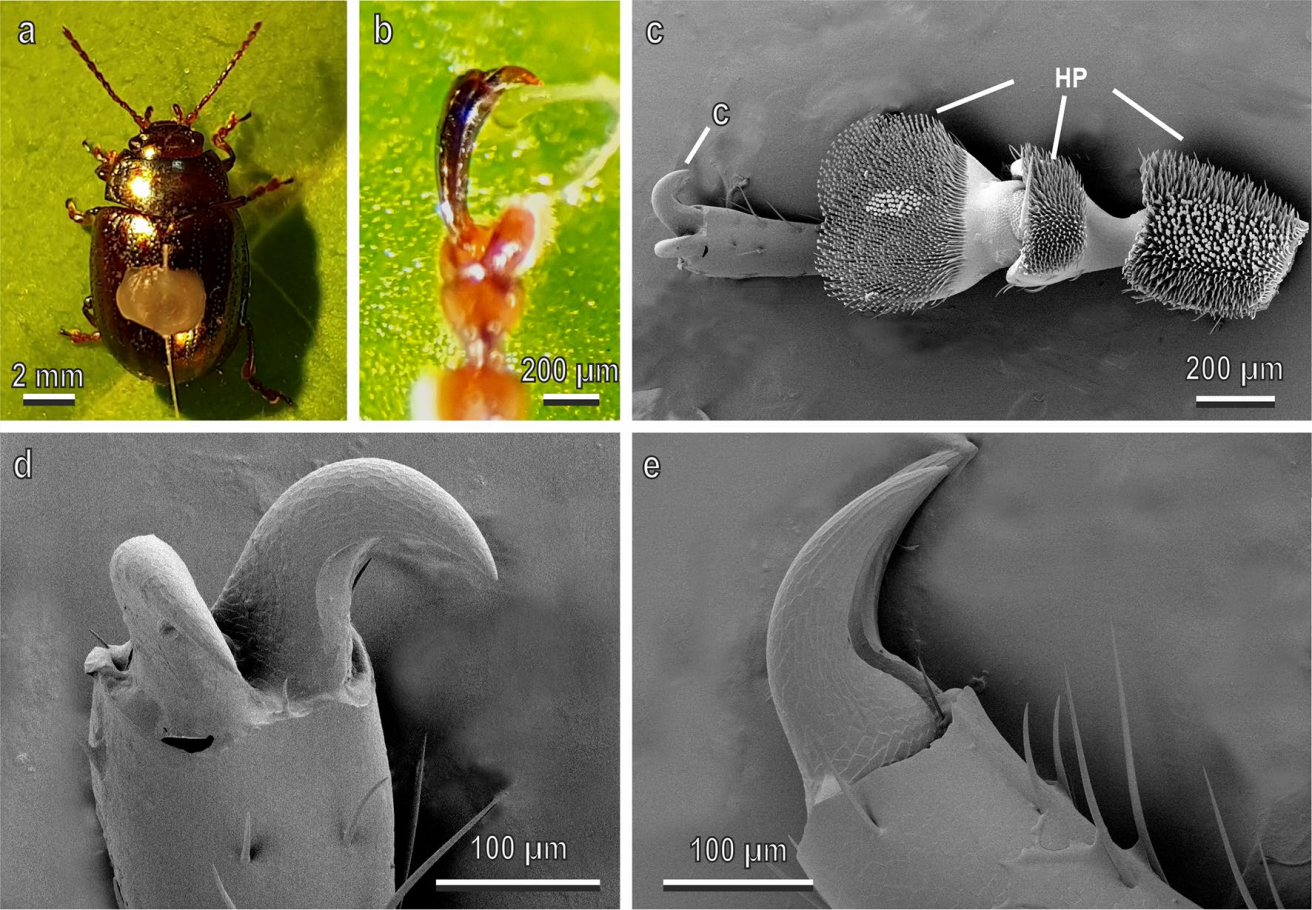
Female (fig. c to be replaced with the female) of *Chrysolina herbacea* under stereomicroscope (**a**, **b**) and tarsal attachment devices in the cryo-SEM (**c**-**e**). **a**, Insect attached to the force sensor by means of the polyethylene thread; **b**, details of the interlocking between the claws and a single trichome of *Cucurbita moschata* leaf; **c**, ventral view of the tarsus with the attachment organs represented by a pair of pretarsal divergent claws (C) and three hairy pads (HP) situated on the ventral side of the first, second, and third tarsal segments; **d**,**e**, details of the two curved divergent claws in frontal (**d**) and lateral (**d**) view

Adults of *C. elaterii* (Fig. 1a) were collected on wild plants of *Ecballium elaterium* (L.) (Cucurbitaceae) in the field close to Perugia (Italy) in June 2020 and reared in the laboratory inside net cages (300 mm × 300 mm × 300 mm) (Vermandel, Hulst, The Netherlands) on plants of *Cucumis melo* L. (Cucurbitaceae) obtained from seeds. Adults of *H. axyridis* (Fig. 2a) (from laboratory culture, Dipartimento di Scienze Agrarie, Alimentari e Ambientali, University of Perugia, Italy) were reared from larvae kept inside net cages (300 mm × 300 mm × 300 mm) (Vermandel, Hulst, The Netherlands) and fed using aphids *Aphis fabae* Scopoli (Hemiptera: Aphididae) reared on young *Vicia faba* L. (Fabaceae) plants. Adults of *C. herbacea* (Fig. 3a) were collected on wild mint plants in the field close to Perugia in June 2020.

The three insect species were kept in a controlled condition chamber (14 h photoperiod, temperature of 23 ± 1 °C, and a relative humidity of 60 ± 10%). Only females were used in the experiments.

### Plants

Two plant species having either visually smooth, shiny leaves (cherry laurel *P. laurocerasus* 'caucasica') or hairy leaves (squash or pumpkin *C. moschata* cv “zucca lunga gigante di Napoli”) were used in the experiments. *C. moschata* was obtained from seeds and *P. laurocerasus* was collected in the field. The adaxial side of the leaf surface was used in the experiments.

### Leaf replicas

Negative moulds of the adaxial side of the leaf surface of *C. moschata* were prepared using the silicone elastomer President light body (PLB; Coltène® Whaledent AG, Altstätten, Switzerland; using the automatic mixing device). PLB was stored in a freezer at  – 18 °C to extend the handling time. A leaf was removed from the plant and silicon elastomer was immediately applied and spread onto the adaxial side of the leaf blade. A Petri dish was gently pressed down upon the silicon elastomer to remove air bubbles and fill folds and asperities of the original substrate. After polymerization (approximately 5 min), the plant surface was carefully peeled off. The negative mould was subsequently filled out with liquid Epon-Araldite resin mixture (Sigma-Aldrich) and polymerized overnight at 60 °C. To hold the liquid resin during polymerisation, a 3 mm-high edge of silicone was created around the negative mould. The hardened positive resin replica of the leaf was removed from the silicone negative mould.

### Cryo-scanning electron microscopy

Samples of (1) adult tarsi of the three coleopteran species, (2) the adaxial side of the tested plant leaves, and (3) resin leaf replicas were studied in a scanning electron microscope (SEM) Hitachi S-4800 (Hitachi High-Technologies Corp., Tokyo, Japan) equipped with a Gatan ALTO 2500 cryo-preparation system (Gatan Inc., Abingdon, UK). For details of sample preparation and mounting for cryo-SEM, see Gorb and Gorb (2009). Insect tarsi and plant leaves were sputter-coated in frozen conditions and the resin replicas in warm conditions with gold–palladium (thickness 10 nm) and examined at 3 kV acceleration in the SEM.

### Force measurements

The friction force of the females of the three tested insect species on the natural and artificial surfaces and the trichome stiffness of natural and artificial substrates were measured using a Biopac force tester (Biopac Systems Ltd, Goleta, CA, USA). For a better interpretation of the results obtained with insects using the Biopac force tester, a centrifugal force tester was applied to measure the attachment ability of females of *H. axyridis* and *C. elaterii* to hydrophilic glass [water contact angle of 32.49 ± 4.17° (mean ± SD)] and the adaxial side of the *C. moschata* leaf. These two different techniques were used, because the Biopac force tester measures the force generated by the insect during locomotion, whereas the centrifugal experiment measures the force generated by the insect when an external force (in the natural situation—e.g., wind, predator) is applied to an insect.

Prior to the force measurements, insects were weighed on a micro-balance (Mettler Toledo AG 204 Delta Range, Greifensee, Switzerland). Experimental insects were anaesthetized with CO_2_ for 60 s and made incapable of flying by gluing their forewings together with a small droplet of melted bee wax. Before starting the experiments, the treated insects were left to recover for 30 min. All the experiments were performed during the daytime at 25 ± 2 °C temperature and 50 ± 5% RH.

*Biopac force tester experiments*. The Biopac force tester consisted of a force sensor FORT-10 (10 g capacity; World Precision Instruments Inc., Sarasota, FL, USA) connected to a data acquisition unit MP 160 (Biopac Systems Ltd, Goleta, CA, USA). Data were recorded using AcqKnowledge 5.0 software (Biopac Systems Ltd, Goleta, CA, USA).

(1) Insect attachment forces. One end of a fishing thread Gel Spun Polyethylene 0.02 mm diameter (Berkley Spirit Lake, Iowa, USA) about 10 cm long was fixed with a droplet of molten wax to the insect thorax. The insect was attached to the force sensor by means of the thread (Figs. 1a, 2a, 3a) and was allowed to move on the test substrate in a direction perpendicular to the force sensor (and parallel to the substrate). The force generated by the insect walking on artificial [hydrophilic glass with water contact angle of 51.71 ± 2.22° (mean ± SD) and resin replicas of leaves with water contact angle of 107.81 ± 8.20° (mean ± SD)] and natural surfaces (the adaxial side of *C. moschata* and *P. laurocerasu*s leaves) was measured. Insect pulled on the plant leaf walking from its proximal to distal portion. Force–time curves were used to estimate the maximal pulling force produced by tethered running insects (traction, friction).

To test the role of claws in the insect attachment ability to trichomes, ablation experiments were carried out. The females were anaesthetized with CO_2_ for 120 s and immobilized with Patafix (UHU Bostik, Milano, Italy) under the stereomicroscope. Claws were removed by cutting off the distal portion of the last tarsal segment with microscissors. Insects used as a control (without ablations) were handled in exactly the same way as the ablated individuals. The insects were left to recover for 24 h before conducting the experiments, to avoid any negative effect due to the manipulations and to the possible bleeding observed just after the ablations. In total, 11 females of *C. elaterii*, 14 females of *H. axyridis*, and 18 females of *C. herbacea* were tested in both conditions, with claws (intact) and without claws (ablated).

**Fig. 4:**
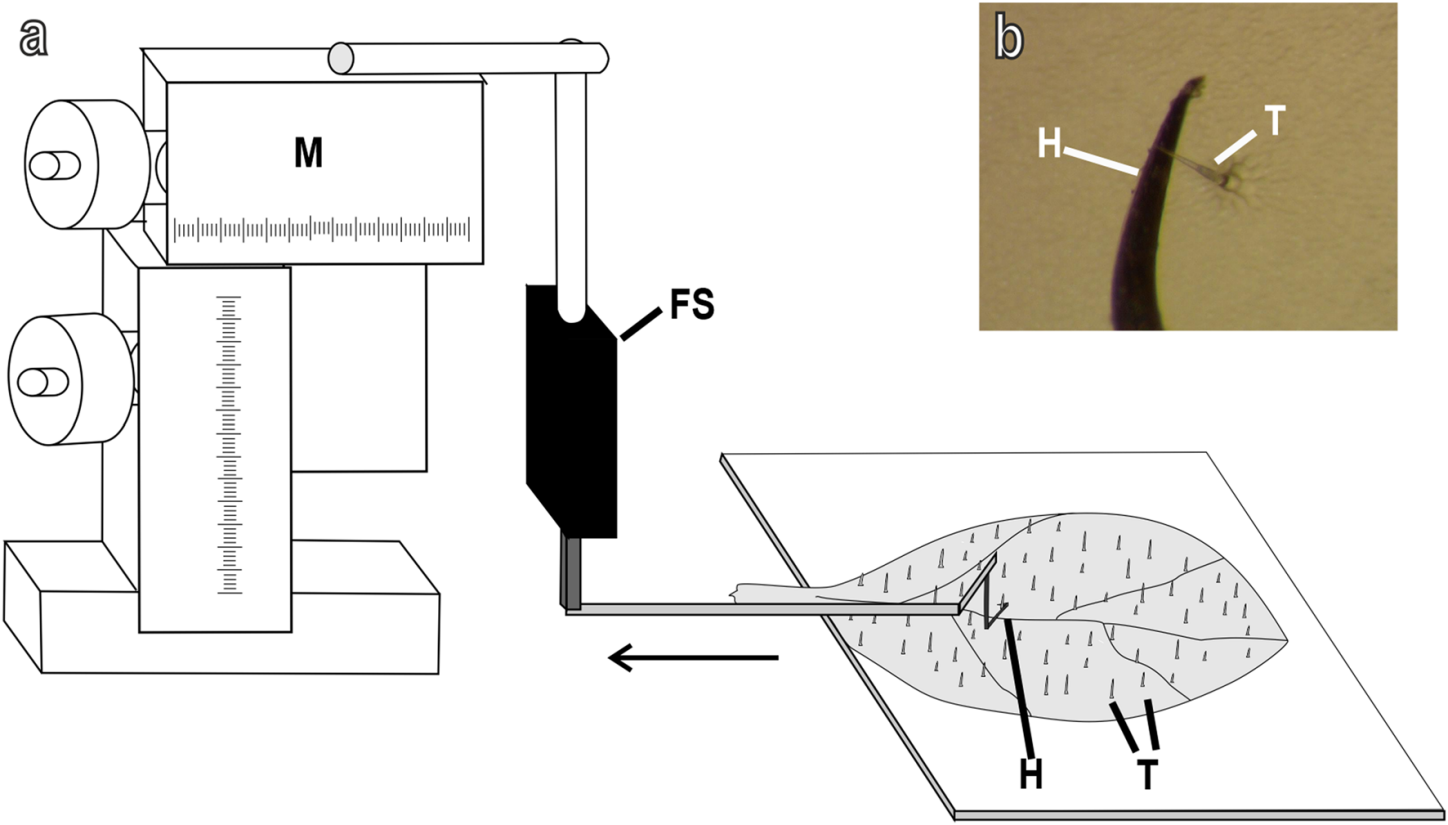
Experimental set-up for testing the trichome stiffness on the adaxial side of the *Cucurbita moschata* leaf. **a**, Metal hook (H) connected to a thin copper lamina attached to the force sensor (FS) fixed to the motorized micromanipulator (M). The hook could contact a single trichome (T) in the middle of its length (see detail under stereomicroscope in **b** and was moved over the leaf from its distal to proximal side (arrow) to push and bend the trichome until loosing contact with it

(2) Trichome stiffness. To measure the trichome stiffness, a special hook (4 mm in its vertical part, 1.4 mm in its horizontal part) made with the tip of a metal pin was connected to a thin copper lamina attached to the force sensor (Fig. 4a). The force sensor was fixed to a motorized micromanipulator (Narishige MMO-203). A leaf of *C. moschata* was fixed with double-sided adhesive tape to a glass plate with the adaxial side facing up. Under visual control with the help of a stereomicroscope Leica MZ6, the hook attached to the sensor was approached to the upper side of the leaf. The hook could contact a single trichome in the middle of its length (Fig. 4b), without touching the leaf surface. The force sensor was moved (speed, 75 µm/s) over the leaf from its distal to proximal side in such a way that the hook could contact the trichome, to push and bend it until finally loose a contact with it.

*Centrifugal force tester experiments*. The centrifugal force tester (Gorb et al. 2001) consists of a metal drum covered by a substrate disc to be tested. The metal drum is driven by a computer controlled motor. Just above the disc, the fibre-optic sensor monitored by the computer is situated. After the positioning of the insect on the horizontal disc, the centrifuge drum started the rotation at a speed of 50 rev min^–1^ (0.883 rev s^−1^). The position of the insect on the drum was monitored using a combination of a focused light beam and a fibre-optical sensor. The drum speed continuously increased until the insect lost its hold on the surface under centrifugal force. The rotational speed at contact loss, the last position of the insect on the drum (radius of rotation), and the insect weight were used to calculate the maximum frictional component of the attachment force. To test the insects on the adaxial side of the *C. moschata* leaf, a portion of the leaf was fixed with tape to the metal drum of the centrifuge. In total, 27 females of *H. axyridis* and 26 females of *C. elaterii* were tested on hydrophilic glass and 22 females of *H. axyridis* and 22 females of *C. elaterii* were tested on the leaf of *C. moschata*.

### Statistical analysis

The stiffness (force of the trichome acting against the moving force sensor) of the non-glandular trichomes of different length (long, medium, and short) covering the adaxial side of *C. moschata* leaf and the resin leaf replicas was analyzed using two-way analysis of variance (ANOVA), considering the surfaces and the trichomes length as factors.

The safety factor (force divided by insect weight) of insect attachment on glass and the normalized friction force (friction force reported as percentage of friction force recorded by each insect on glass) on the different surfaces (the adaxial leaf surface of *C. moschata*, its resin replica, and the adaxial leaf surface of *P. laurocerasus*) of the females of the three insect species with claws and without claws were analyzed using two-way repeated-measures analysis of variance (ANOVA), considering the insect condition (with and without claws) and the insect species as main factors. When necessary, after all the ANOVA tests, for significant factors, Tukey unequal N HSD post hoc test for multiple comparisons between means was performed (Statistica 6.0, Statsoft Inc. 2001). In the centrifugal force tester experiments, the Mann–Whitney *U* test was used for the comparison between *H. axyridis* and *C. elaterii*, both for the friction force and safety factor. Before the parametric analysis, all the data were subjected to Box–Cox transformations, to reduce data heteroscedasticity (Sokal and Rohlf 1998).

## Results

### Morphological characterization of claws of adult beetles, plant surfaces, and their resin replicas

The tarsi of *C. elaterii* (Fig. 1) are composed of four segments. The first and the second ones bear two ventral hairy pads with numerous “tenent setae”, while the fourth bears the pretarsal claws (Fig. 1c); each claw is composed of two bifid sub-claw with a basal tooth (bifid dentate or bifid appendiculate claws) separated from the main portion of the structure by a deep cleft (Fig. 1d,e). Such a cleft between the basal tooth and the bifid portion of the claw is constituted of an angle measuring about 19°. The cleft separating the bifid claw measures about 15°.

The tarsi of *H. axyridis* (Fig. 2) are also tetramerous. The first and the second segments have the two hairy pads with numerous tenent setae, while the fourth one bears the pretarsal claws (Fig. 2c). Each claw shows a wide basal tooth (dentate or appendiculate claws) separated from the main portion of the claw by a cleft (Fig. 3c–e). The cleft between the basal tooth and the claw is very deep with an angle of about 11°.

The tarsi of *C. herbacea* (Fig. 3) are composed of five tarsal segments. Tarsomeres 1–3 bear hairy pads with numerous tenent setae on the ventral side, while the fifth segment is equipped with the pretarsal claws (Fig. 3c), constituted of two curved divergent claws (Fig. 3c–e).

**Fig. 5:**
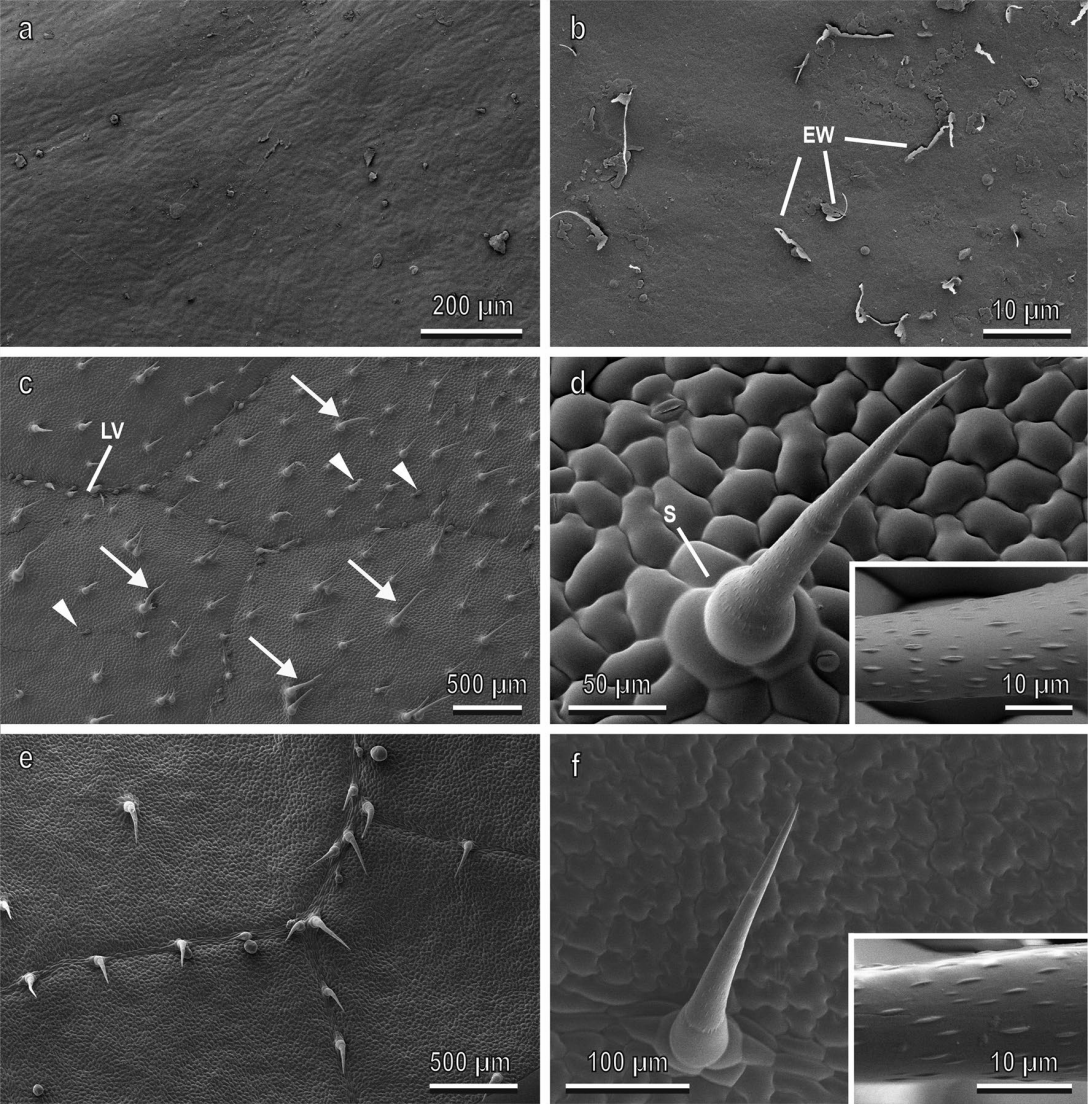
Adaxial side of the tested leaf surfaces (**a**–**d**) and resin replica of the adaxial side of *Cucurbita moschata* leaf in the cryo-SEM (**e**,**f**). **a** Leaf surface of *Prunus laurocerasus* lacking trichomes; **b** details of the leaf surface with loosely distributed epicuticular wax projections (*EW*); **c**, Adaxial leaf surface of *Cucurbita moschata* with numerous non-glandular (arrows) and glandular (arrow head) trichomes. *LV* leaf veins; **d**, details of a multicellular non-glandular trichome with its socket (S). Inset shows a detail of the trichome sculpturing by numerous microscopic ellipsoid knobby protrusions; **e**, **f**, highly faithful reproduction of the morphological details of the natural leaf surface shown in (**c**) and (**d**). Inset indicates the minute detail of the surface sculpturing of non-glandular trichomes clearly visible in the resin replicas

The adaxial side of the *P. laurocerasus* leaf lamina is rather smooth although slightly uneven, without trichomes (Fig. 5a). The surface is covered with 2D epicuticular wax film or layer bearing very flat, plate-like surface irregularities and sparsely scattered 3D epicuticular wax projections (membraneous platelets) (Fig. 5b).

The adaxial leaf surface of *C. moschata* is regularly covered by numerous non-glandular and glandular trichomes (Fig. 5c), which are situated on both the leaf veins and areas between the veins. Non-glandular trichomes (Fig. 5d) are multicellular, uniseriate, with multicellular sockets at the base, non-branched, and cone-shaped with sharp tips. Their surface bears a sculpturing created by numerous microscopic ellipsoid knobby protrusions (inset of Fig. 5d). These slightly inclined or bent trichomes are responsible for a rather uniform and dense (69.6 ± 5.9 trichomes per 1 mm^2^) leaf pubescence and have a greatly variable length. In particular, three size classes of non-glandular trichomes (short, medium, and long) can be distinguished according to their length and diameter, as reported in Table 1. Glandular capitate trichomes of two types—short-stalked and long-stalked (types I and II according to Kolb and Müller 2004)—are usually shorter and distributed much more sparsely than non-glandular ones. Also stomata are scattered over the adaxial leaf surface.

**Table 1:** Morphometrical variables (in µm) of the non-glandular trichomes located on the adaxial side of the *Cucurbita moschata* leaf categorized on the basis of their length and diameter (mean ± SE) in three size classes (long, medium, and short)

	Type	Length	Diameter	*n*
Leaves	Long	869.56 ± 61.06	48.73 ± 3.59	6
	Medium	467.80 ± 28.78	28.85 ± 3.09	5
	Short	262.60 ± 24.33	19.75 ± 2.50	5
Resin replica	Long	558.33 ± 27.84	25.08 ± 2.75	5
	Medium	327.40 ± 22.53	18.60 ± 2.78	5
	Short	209.15 ± 14.95	14.53 ± 1.55	5

Detailed analysis of the resin replicas of the adaxial side of the leaf of *C. moschata* (Fig. 5e–f) revealed a highly faithful reproduction of the morphological details of the natural leaf surface. The trichomes in the replicas shows exactly the same morphology described in the leaves. Even a minute detail, such as the surface sculpturing of non-glandular trichomes by numerous microscopic ellipsoid knobby protrusions, is clearly visible in the resin replicas (inset of Fig. 5f).

**Fig. 6:**
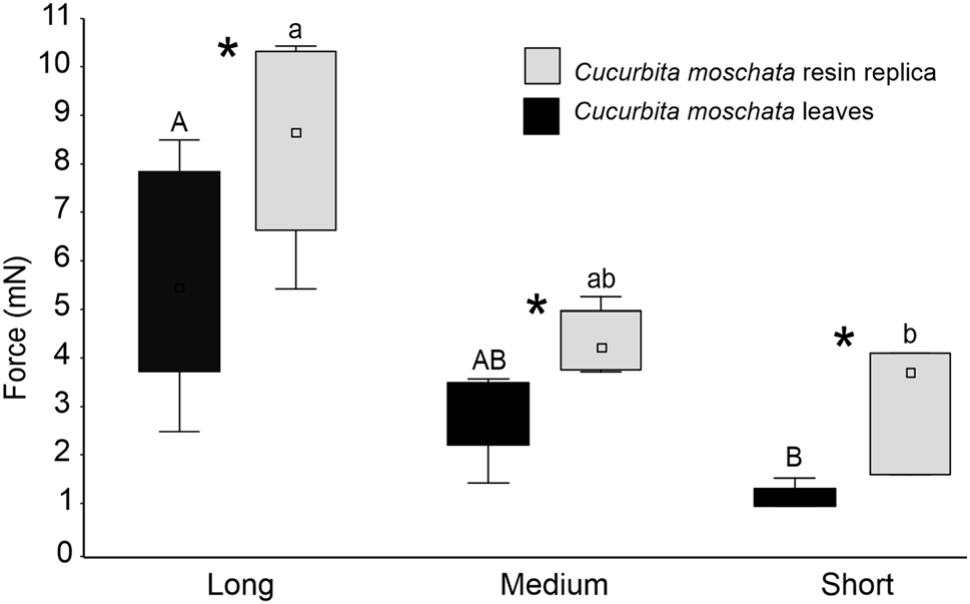
Stiffness (force of the trichome acting against the moving force sensor) of the non-glandular trichomes of different length (long, medium, and short, see Table 1) covering the adaxial side of *Cucurbita moschata* leaf and the resin leaf replicas. Boxplot shows the interquartile range and the median (square), whiskers indicate 1.5 × interquartile range. Boxplots with different upper case letters and lower case letters, respectively, are significantly different at *P* < 0.05. In the comparison between the leaf surface and resin leaf replicas, asterisk (*) means significant difference at *P* < 0.05 (Tukey unequal N HSD post hoc test, two-way ANOVA)

Force experiments revealed that for all the measured trichomes (short, medium, and long), the stiffness was higher in the trichomes of the resin replicas, if compared with those present on the intact leaf of *C. moschata* (Fig. 6). The stiffness was different among the three size classes of trichomes (surfaces: F = 17.0, d.f. = 1, 21, *p* = 0.0005; size classes: F = 25.0, d.f. = 2, 21, *p* < 0.0001; surfaces × size classes: F = 0.4, d.f. = 2, 21, *p* = 0.6917).

### Insect friction force on different substrates

**Fig. 7:**
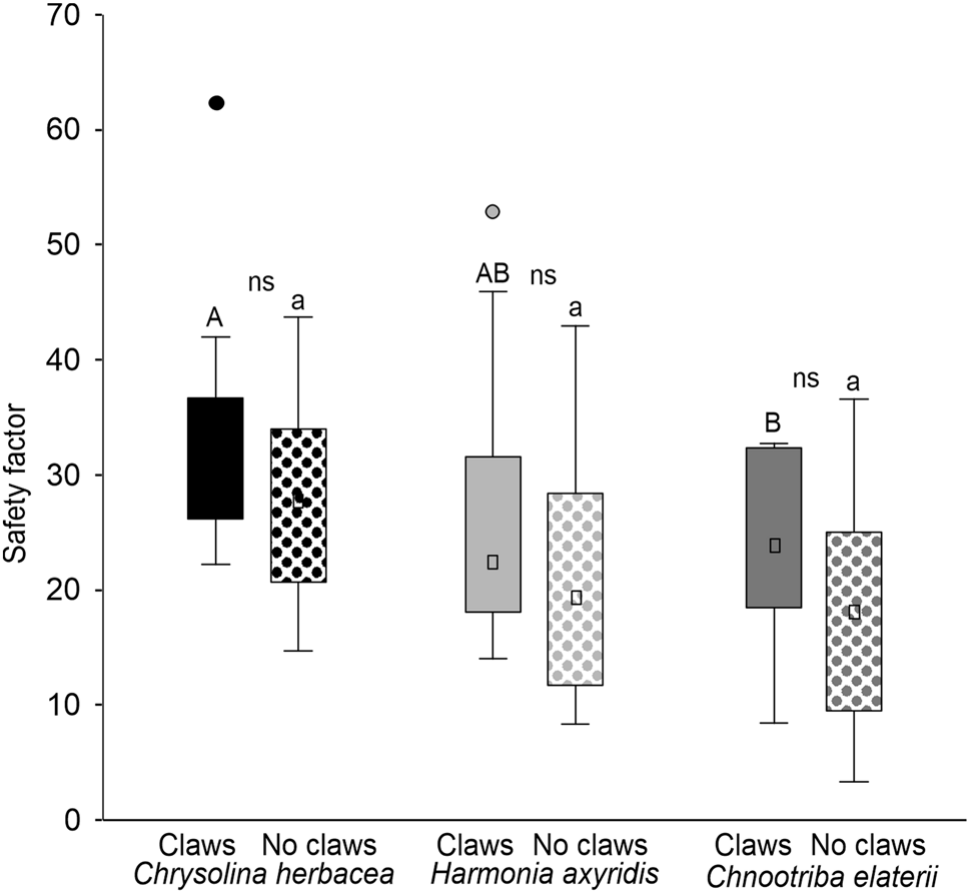
Safety factor (force divided by insect weight) of the females of *Chrysolina herbacea*, *Harmonia axyridis*, and *Chnootriba* *elaterii* pulling on glass in the different conditions, with claws (intact) and without claws (ablated). Boxplots show the interquartile range and the medians, whiskers indicate 1.5 × interquartile range and “°” shows outliers. Boxplots with different upper case letters and lower case letters, respectively, are significantly different at *P* < 0.05. In the comparison between insects with claws and without claws, ns means not significantly different (Tukey unequal N HSD post hoc test, repeated-measures two-way ANOVA)

Force experiments with the females of the three beetle species pulling on glass in different conditions (intact and ablated) revealed that in all the tested species, there was no significant difference in the attachment ability between intact and ablated insects (Fig. 7). In intact insects, the highest safety factor was recorded in *C. herbacea* and the lowest one in *C. elaterii*, while *H. axyridis* showed intermediate safety factor value (insect conditions: F = 2.2, d.f. = 1, 36, *p* = 0.1454; species: F = 7.0, d.f. = 2, 36, *p* = 0.0028; insect conditions × species: F = 0.8, d.f. = 2, 36, *p* = 0.4416).

**Fig. 8:**
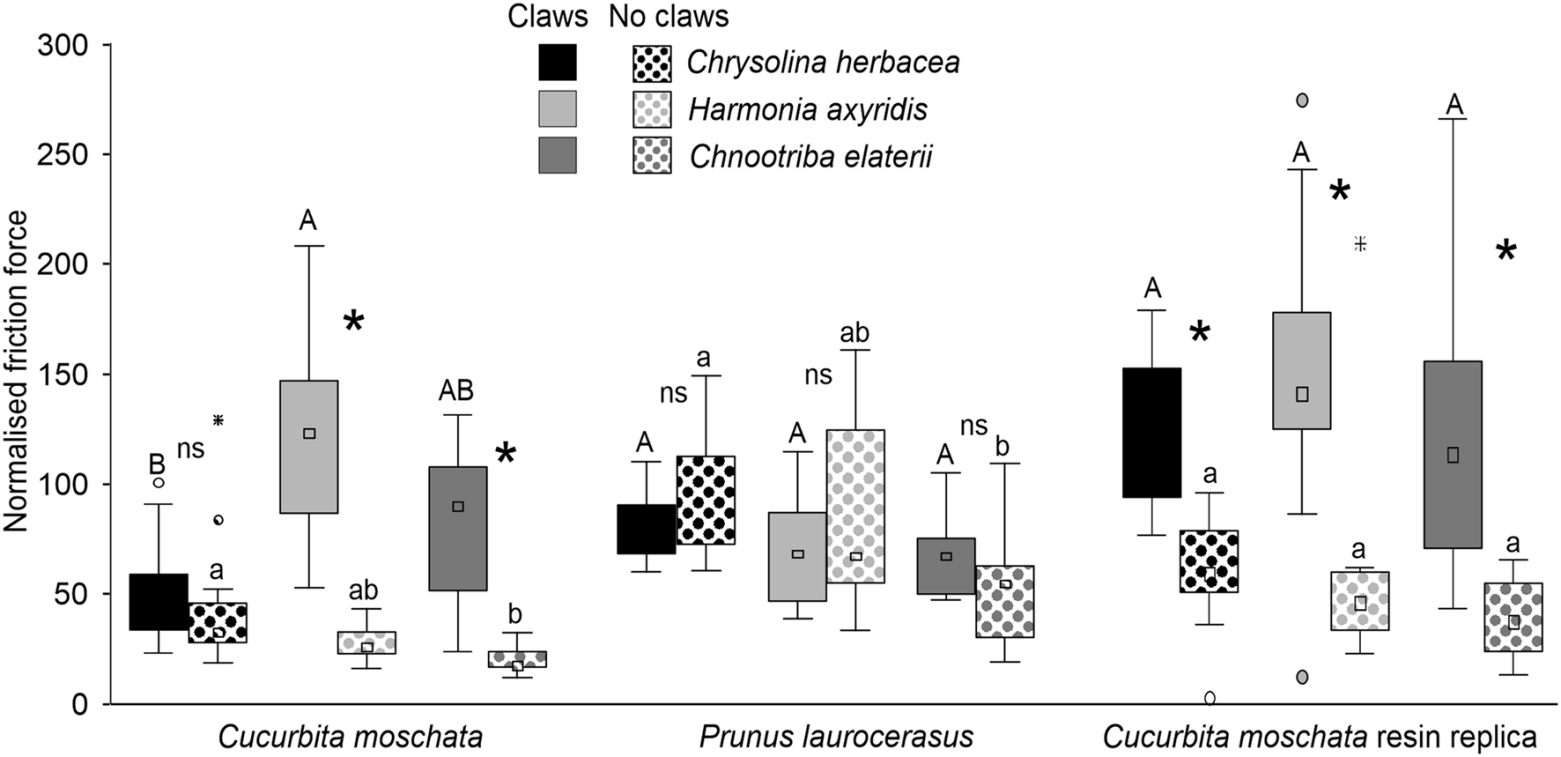
Normalized friction force of the females of *Chrysolina herbacea*, *Harmonia axyridis*, and *Chnootriba elaterii* pulling on the adaxial side of the *Cucurbita moschata* leaf (hairy surface), on its resin replica and on the adaxial side of the *Prunus laurocerasus* leaf (smooth surface) in different conditions of their attachment system (with claws (intact) and without claws (ablated)). Boxplots show the interquartile range and the medians, whiskers indicate 1.5 × interquartile range and “°” shows outliers. For each surface, boxplots with different upper case letters and lower case letters, respectively, are significantly different at *P* < 0.05. In the comparison between insects with claws and without claws, asterisk (*) means significant difference at *P* < 0.05 and ns means not significant difference (Tukey unequal N HSD post hoc test, repeated-measures two-way ANOVA)

Force experiments with females of the three tested species pulling on the adaxial side of the *C. moschata* leaf (hairy surface), on its resin replica and on the adaxial side of the *P. laurocerasus* leaf (smooth surface) in the different conditions (intact and ablated) (Fig. 8) revealed that on the smooth surface, there was no significant difference in the normalized friction force (% of friction force on glass) of intact and ablated insects in all the three insect species. Moreover, the attachment ability of intact insects on the smooth leaf surface was similar in the three species and slightly reduced, if compared to that on glass (insect conditions: F = 0.5, d.f. = 1, 34, *p* = 0.4959; species: F = 9.3, d.f. = 2, 34, *p* = 0.0006; insect conditions × species: F = 2.8, d.f. = 2, 34, *p* = 0.0730) (Fig. 8). On the hairy surface of the leaf of *C. moschata*, a very high force was recorded for *H. axyridis* and *C. elaterii*, while a very low one for *C. herbacea*. Such a high attachment ability of *H. axyridis* and *C. elaterii* was strongly reduced in insects without claws, while there was no significant difference in the attachment ability between intact and ablated insects in *C. herbacea* (insect conditions: F = 144.4, d.f. = 1, 34, *p* < 0.0001; species: F = 5.1, d.f. = 2, 34, *p* = 0.0084; insect conditions × species: F = 25.7, d.f. = 2, 34, p < 0.0001) (Fig. 8). On the resin replica of the adaxial side of the *C. moschata *leaf, a very high force (higher than that on glass) was recorded in intact insects in all the three tested species, while the attachment ability was strongly reduced in ablated insects (insect conditions: F = 72.4, d.f. = 1, 34, *p* < 0.0001; species: F = 0.8, d.f. = 2, 34, *p* = 0.4486; insect conditions × species: F = 1.2, d.f. = 2, 34, *p* = 0.3184) (Fig. 8).

**Fig. 9:**
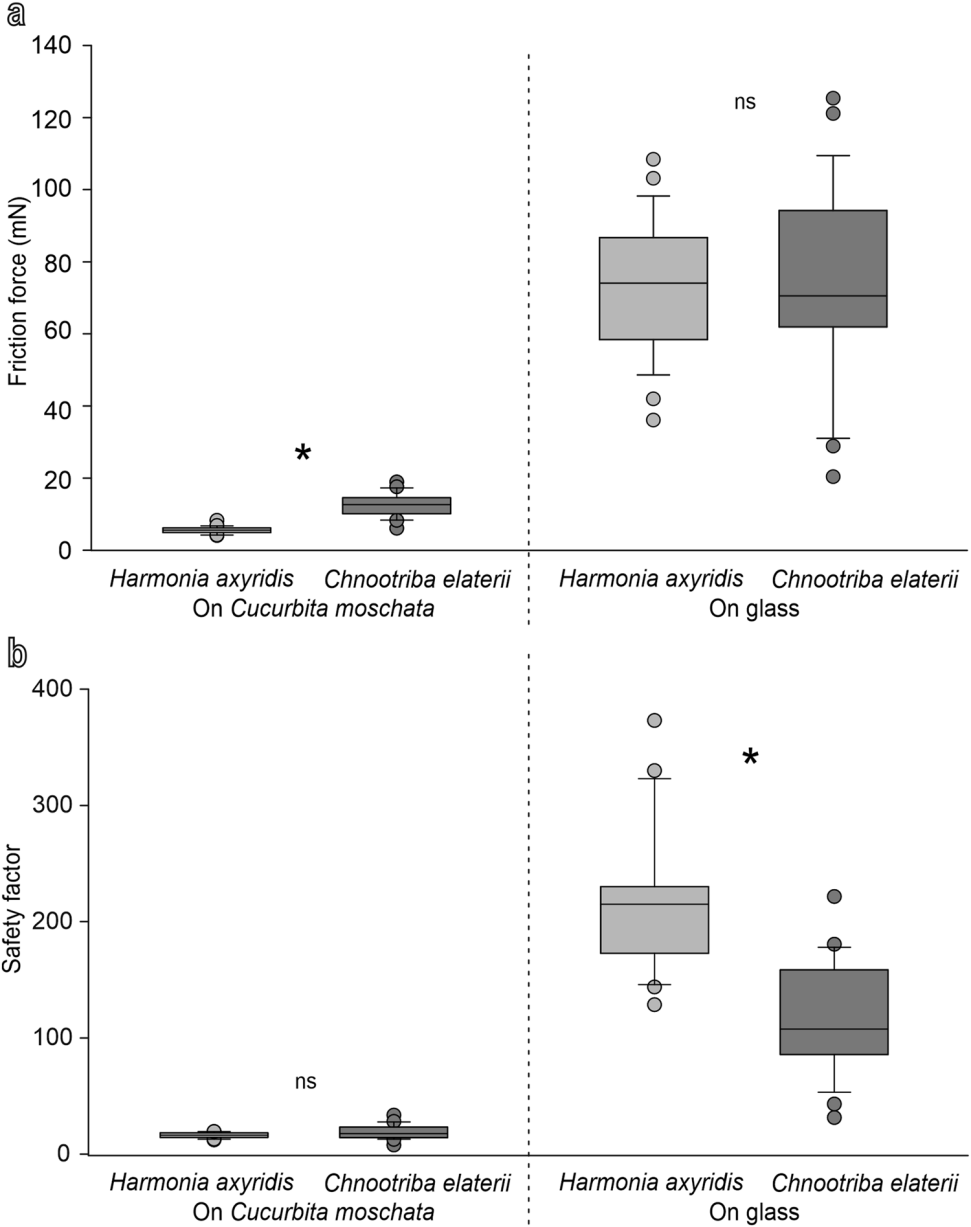
Friction force (**a**) and safety factor (force divided by insect weight) (**b**) of the females of *Harmonia axyridis* and *Chnootriba elaterii* obtained in centrifugal force tester experiments on the adaxial side of the *Cucurbita moschata* leaf and on hydrophilic glass. Boxplots show the interquartile range and the medians, whiskers indicate 1.5 × interquartile range and “°” shows outliers. In the comparison between the two insect species, asterisk (*) means significant difference at *P* < 0.05 and ns means not significant difference (Mann–Whitney *U* test)

In the centrifuge experiments performed with the females of *H. axyridis* and *C. elaterii* on hydrophilic glass, there was no significant difference in the friction force of the two species (U = 326; T = 727; N = 26, 27; *p* = 0.663), while in consideration of the higher weight of *H. axyridis*, its safety factor was higher than that of *C. elaterii* (U = 646.5; T = 406.5; N = 26, 27; *p* < 0.001) (Fig. 9). In the same experiments performed on the adaxial side of the *C. moschata* leaf, the friction force of *C. elaterii* was higher than that of *H. axyridis* (U = 7; T = 730; N = 22, 22; *p* < 0.001), while in consideration of the higher weight of *C. elaterii*, there was no significant difference in the safety factor between the two species (U = 172; T = 565; N = 22, 22; *p* = 0.103) (Fig. 9).

## Discussion

The data reported in the present study revealed that claws with different shape (bifid dentate, dentate, or divergent), observed at the adult stage of three different beetle species represented by the phytophagous ladybird *C. elaterii*, the predaceous ladybird *H. axyridis* and the phytophagous leaf beetle *C. herbacea*, have different involvement in insect attachment to plant hairy surfaces, such as the leaf of *C. moschata*, characterized by a dense pubescence formed mainly by non-glandular/non-branched straight trichomes. In particular, the dentate claws of the two coccinellid species (*C. elaterii* and *H. axyridis*) allow a higher attachment ability to the adaxial side of the hairy leaf of *C. moschata*, if compared to the divergent claws without basal tooth of *C. herbacea*. Such a high attachment ability of the two coccinellid species (for *H. axyridis* higher than that recorded for smooth glass) is due to the presence of dentate claws: in ablated insects, the attachment ability of *C. elaterii* and *H. axyridis* is strongly reduced, while there is no difference in the attachment ability of *C. herbacea* with or without claws to *C. moschata*.

Interestingly, when we tested the three beetle species on the resin replica of *C. moschata* leaf, we could record a high attachment ability (higher than that recorded on glass) also in *C. herbacea* and the claws ablation significantly reduced the insect attachment in all the tested species. These results, different from those recorded on the natural hairy surface (leaf), are probably related to the different trichome stiffness characterizing natural and artificial surfaces. Indeed, when we compared the trichome stiffness of the resin replica with that of the *C. moschata* leaf, we could record much higher resistance to trichome bending in resin replicas for the trichomes of different sizes (short, medium, and long).

Non-glandular trichomes of different plant species can have different stiffness, but they tend to be soft (mobile), especially in their basal portion, as shown in the hooked trichomes of *P. vulgaris* leaf (Szyndler et al. 2013; Rebora et al. 2020a) or *Galium aparine* L. (Rubiaceae) (Gorb et al. 2002). Indeed, EDX analysis and SEM observations of the hooked trichomes in *P. vulgari*s revealed the higher amount of silicon in the distal (hooked) part of the trichome (Szyndler et al. 2013) and the lower amount in its basal portion presumably making it more flexible (Rebora et al. 2020a). Some other plant trichomes contain a set of thin-walled cells at the base, building together the more flexible region of the trichome (Gorb et al. 2002). Such a basal region could serve as a flexible micro-joint, able to twist without breaking when bent. The flexible basal and medial portions of the trichome require some special tool to interlock without slipping and the basal tooth of the claw in the two coccinellid species, with the deep cleft, allows a firm grip with the trichome (Figs. 1b, 2b), which can bend, when pulled, keeping the interlocking with the insect claw. A similar situation has been described in the mirid bug *D. errans* typically living on pubescent plants, where observations under SEM revealed that one part of the paired claws can interlock with the middle region of flexible plant trichomes using the border of the basal claw plate and the ventro-distal claw surface (Voigt et al. 2007). The basal claw plate described in *D. errans* could have a function analogous to that of coccinellid basal tooth.

On the other hand, the tendency to develop claws with numerous clefts has been described in different insect species, which show serrate or pectinate claws to attach to highly flexible surfaces. For example, *Craterina pallida* Latreille (Diptera: Hippoboscidae), an avian ectoparasite, attaches to the bird feathers using large tridentate claws (Petersen et al. 2018) and the bee louse *Braula coeca* Nitzsch (Diptera: Braulidae) has tiny comb-like claws and attaches to hairs on the honey bee thorax (Büscher et al. 2021). On the contrary, the simple divergent claws of *C. herbace*a tend to slip over the flexible trichomes (which bend, when pulled, owing to their flexibility), resulting in a very weak contact formation and overall attachment force. Such problem disappears, when *C. herbacea* walks on resin leaf replicas with trichomes of the original shape, but characterized by a higher stiffness. This is in agreement with what has been previously observed in *D. errans* in traction force experiments in relation to trichome morphometry (Voigt et al. 2007), where longer and thicker trichomes were found to enhance bug traction force, probably in the context of the trichome bending stiffness.

Similar to the results previously obtained in different other studies (e.g., Bullock and Federle 2011), also in the beetle species here tested, the involvement of claws is not important in insect attachment to smooth surfaces, since no significant difference was detected in the attachment ability between intact or ablated insects of the three species measured on smooth artificial or natural surfaces, such as glass or the leaf surface of *P. laurocerasus*. The lack of difference in the attachment ability to smooth surfaces of intact or ablated insects allows us to exclude the effect due to any possible damage to the unguitractor tendon complex during claw amputation.

The slight reduction in attachment ability of both intact and ablated insects on the natural non-hairy plant surfaces, if compared to glass, is probably related to the presence of some wax projections on the leaf surface of *P. laurocerasus*, since 3D wax projections on plant surfaces strongly interfere with insect attachment ability (e.g., Eigenbrode 2004; Voigt et al. 2007; Gorb and Gorb 2017; Salerno et al. 2018a; Rebora et al. 2020b).

**Fig. 10:**
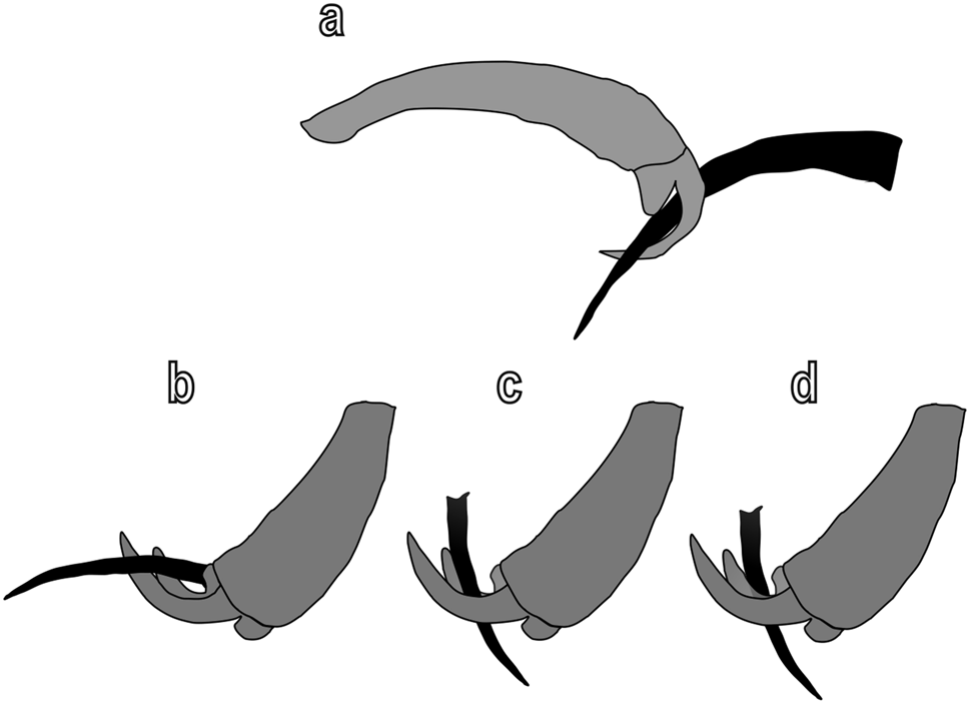
Schematic reconstruction of the trichome interlocking with the dentate claws of *Harmonia axyridis* (**a**) and *Chnootriba elaterii* (**b**–**d**) based upon behavioral observations. Note that the deep cleft between the tooth and the claw allows a firm grip with the trichome. The presence of two clefts in each claw of *C. elaterii* potentially represents two clamping regions that can interlock the trichome in different areas and with different orientation

Our traction force experiments suggest that the dentate claws of the two coccinellid species could represent an adaptation to attach to hairy plant surfaces, but do not clarify the morpho-functional differences between the bifid dentate claws of *C. elaterii* and the dentate claws of *H. axyridis*. The presence of two clefts in each claw of *C. elaterii*, potentially representing two clamping regions that can interlock with the trichomes in different areas and with different orientation (Fig. 10), should ensure higher attachment ability to hairy surfaces in *C. elaterii* compared to *H. axyridis*, which possesses a single cleft in the claw. However, the data of the present study reveal similar attachment ability in the two species, with a slightly higher safety factor in the second species. A possible explanation of these results is the different habits of the two species, with the predatory ladybug *H. axyridis* revealing a higher safety factor (not only on hairy surfaces, but also on smooth glass) due to the higher muscular force, if compared to a phytophagous species *C. elaterii*. In addition, *H. axyridis* tends to be more active, when walking on *C. moschata*, probably looking for prey, while *C. elaterii* tends to rest on its host plants. This is the reason, why we performed centrifugal force tester experiments to measure the force generated by the insect, when an external force is applied to it (different from that measures with walking and pulling insects recorded with the Biopac force tester). In the centrifuge experiments, the forces and safety factors generated by the two species on glass (due to hairy pads) were similar to those recorded in traction force experiments, but the forces and safety factors obtained on the hairy plant surface (due to claws with different shape) were highly reduced because of the insect difficulty to find the right interlocking with the plant trichomes, when exposed to an external force. In any case, the friction force was higher in *C. elaterii* than in *H. axyridis*, thus making us hypothesize that the bifid dentate claws of *C. elaterii* could represent a possible adaptation of the phytophagous ladybird to its host plant species from the family Cucurbitaceae, which are typically characterized by hairy surfaces. *H. axyridis*, as a predator, is able to attach well on different plants were prey can be found and is less specialized in its attachment structures than a phytophagous ladybird dependant on a specific plant.

In conclusion, the data here presented reveal that plant trichomes can enhance insect attachment to plant surface compared with smooth glass by increasing the friction force, but this effect is directly related to their stiffness. To effectively grasp soft trichomes, insects evolved special claws-associated structures, such as the dentate claws observed in Coccinellidae. Especially, effective adaptations to attach to the hairy plant surfaces are present in the claws of phytophagous ladybirds closely associated with hairy species from the family Cucurbitaceae.
